# Enrichment of intersubtype HIV-1 recombinants in a dual infection system using HIV-1 strain-specific siRNAs

**DOI:** 10.1186/1742-4690-8-5

**Published:** 2011-01-13

**Authors:** Yong Gao, Measho Abreha, Kenneth N Nelson, Heather Baird, Dawn M Dudley, Awet Abraha, Eric J Arts

**Affiliations:** 1 Division of Infectious Diseases, Department of Medicine, Case Western Reserve University, 10900 Euclid Ave, Cleveland, Ohio 44106, USA; 2 Department of Molecular Biology and Microbiology, Case Western Reserve University, 10900 Euclid Ave, Cleveland, Ohio 44106, USA

## Abstract

**Background:**

Intersubtype HIV-1 recombinants in the form of unique or stable circulating recombinants forms (CRFs) are responsible for over 20% of infections in the worldwide epidemic. Mechanisms controlling the generation, selection, and transmission of these intersubtype HIV-1 recombinants still require further investigation. All intersubtype HIV-1 recombinants are generated and evolve from initial dual infections, but are difficult to identify in the human population. In vitro studies provide the most practical system to study mechanisms, but the recombination rates are usually very low in dual infections with primary HIV-1 isolates. This study describes the use of HIV-1 isolate-specific siRNAs to enrich intersubtype HIV-1 recombinants and inhibit the parental HIV-1 isolates from a dual infection.

**Results:**

Following a dual infection with subtype A and D primary HIV-1 isolates and two rounds of siRNA treatment, nearly 100% of replicative virus was resistant to a siRNA specific for an upstream target sequence in the subtype A envelope (*env*) gene as well as a siRNA specific for a downstream target sequence in the subtype D *env *gene. Only 20% (10/50) of the replicating virus had nucleotide substitutions in the siRNA-target sequence whereas the remaining 78% (39/50) harbored a recombination breakpoint that removed both siRNA target sequences, and rendered the intersubtype D/A recombinant virus resistant to the dual siRNA treatment. Since siRNAs target the newly transcribed HIV-1 mRNA, the siRNAs only enrich intersubtype env recombinants and do not influence the recombination process during reverse transcription. Using this system, a strong bias is selected for recombination breakpoints in the C2 region, whereas other HIV-1 env regions, most notably the hypervariable regions, were nearly devoid of intersubtype recombination breakpoints. Sequence conservation plays an important role in selecting for recombination breakpoints, but the lack of breakpoints in many conserved env regions suggest that other mechanisms are at play.

**Conclusion:**

These findings show that siRNAs can be used as an efficient in vitro tool for enriching recombinants, to facilitate further study on mechanisms of intersubytpe HIV-1 recombination, and to generate replication-competent intersubtype recombinant proteins with a breadth in HIV-1 diversity for future vaccine studies.

## Background

Recombination between two genetically distinct isolates of the same retrovirus species was first described in the 1970s [1,2]. Retroviral recombination originates from two different virus isolates co-infecting a single cell and the production of heterodiploid retrovirus particles [3]. Upon de novo cell infection, reverse transcriptase jumps between the two heterologous genomes during (-) or (+) strand DNA synthesis and creates a chimeric proviral genome. HIV-1 recombination is very common during infection and may be a major evolutionary mechanism responsible for shuffling of nucleotide substitutions introduced by the error-prone reverse transcriptase [4,5]. As a consequence, recombination accelerates intrapatient HIV-1 diversity as well as evolution from the founder virus. Within the epidemic, circulation of HIV-1 mosaics encoded by chimeric genomes indicates that an HIV-1 recombination must have arisen following a primary infection with two founder viruses of different subtypes or due to a superinfection with a different subtype virus [6-8]. The consequences of intersubtype recombination within dual/superinfected individual can be profound and can lead to the immediate selection of unique recombinant forms (URFs) or subsequent transmission of stable circulating recombinant forms (CRFs) [9]. Based on partial or full genome sequencing of HIV-1 isolates from around the world, at least 20% of the 33 million infected humans harbor an intersubtype URF or CRF [6,10,11]. For example, in East Africa, intersubtype A/D, A/C, and D/C recombinant forms are almost as common as the parental subtype A, C, and D[8]. These URFs and CRFs have the potential to foil vaccine strategies based on single subtypes and even lead to rapid drug resistance.

The mechanisms and selection of intersubtype HIV-1 recombinations in humans have been difficult to study due to the rare occurrence of dual infection or superinfection with two of more HIV-1 isolates. Intersubtype HIV-1 recombinants can be generated in tissue culture using dual infections, but the parental strains generally dominate or out-compete the very few functional recombinant forms [12,13]. Our previous studies described a marked decrease in the overall recombination rates in the multiple cycle tissue culture assays (range from 0.25 to 3.4%) than in single cycle (4-17%) or in vitro (6-30%) systems, where recombinants are subject to selection for replicative capacity [13]. Recombination rates further decrease when utilizing divergent primary HIV-1 isolates of different subtypes [13]. For example, recombination frequency between two subtype A viruses was significantly greater than between a subtype A and D virus [13]. To date the majority of studies on HIV-1 recombination have utilized defective retroviral constructs that can recombine in select genomic regions (introduced by cloning), but in this system, there are no functional or replication requirements for the generation of these recombinants [14-16]. We have employed primary HIV-1 isolates in dual infection studies to determine the frequency of intra- and inter-subtype recombination and to map crossover sites [12,13,17]. However, in these studies, the HIV-1 recombinants may or may not be functional and only represent 0.5 to 3% of the virus population [12,13,17]. In this study, the use of HIV-1 strain-specific siRNA can effectively enrich for recombinants by eliminating the parental virus populations, which would otherwise dominate a dually infected culture. Previous studies have enriched for HIV-1 recombinants between drug resistant mutations by using two different drug resistant variants and culturing a dual infection in the presence of the two antiretroviral drugs [18,19]. As described below, our recombination system differs from previous studies in that nearly any two divergent HIV-1 strains can be recombined (not just drug resistant variants) and in any genomic region flanked by divergent sequences for different siRNA targets.

RNA interference (RNAi) was first described in nematodes as a specific mechanism to regulate gene expression at post-transcriptional level [20]. In the case of long dsRNA molecules, Dicer cleaves the RNA into 21bp dsRNAs, termed small interfering RNA (siRNA) duplexes; one strand of which is then incorporated into a ribonuclease-containing RNA induced silencing complex (RISC) [21]. The siRNA within RISC then guides the complex to specifically target mRNAs [22]. Once RISC has bound a mRNA bearing a matched sequence, the mRNA can be cleaved. Sequence-specific anti-HIV-1 effects have been observed following the introduction of synthetic siRNAs (ssiRNAs) via transfection into HIV-1-permissive cells, or via endogenous expression of 21-23 nt transcripts (psiRNAs) or hairpin RNAs (pshRNAs) from DNA plasmids [23-25]. Aside from its use as a molecular tool, there is considerable interest in the development of RNAi as a possible treatment or prevention strategy for HIV-1 infections as well as other viral diseases [26].

**Figure 1:**
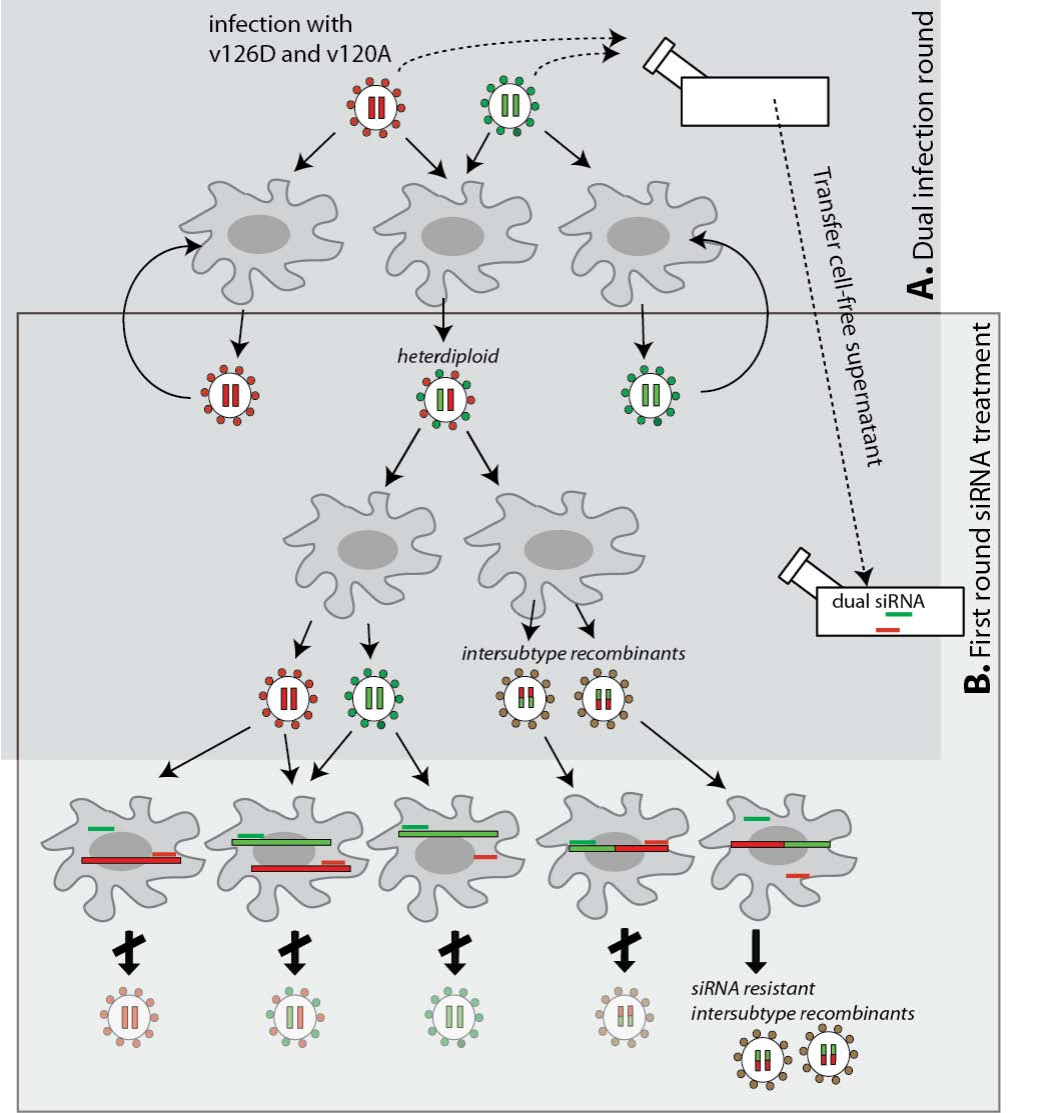
**Schematic illustration on how siRNAs may enrich for intersubtype HIV-1 recombinants**. HIV-1 v120-A and v126-D were used for mono- or dual-infection of U87.CD4.CXCR4 cells with equal or different MOI in this study. This figure illustrates how two siRNAs specific for the 5' end of v120-A *env *and 3' end of v126-D *env *might enrich for v126-D/v120-A recombinants after an initial dual infection and then propagation with siRNAs. During the initial dual infection (panel **A**), v120-A and v126-D are produced from monoinfected cells while a co-infected cell can produce a heterodiploid virus particle containing an RNA genome from each virus. If the heterodiploid virus infects and replicates in the initial dually infected cultures, a v120-A/v126-D or v126-D/v120-A recombinant virus can then be produced in the next round of infection but it is likely in lower abundance than the parental viruses. The five general types of virus, produced from initial dual infection, are then used to infect fresh cells treated with siRNAs (Panel **B**). In this round, siRNA120a primarily inhibits HIV-1 v120-A whereas siRNA126a would inhibit v126-D. In addition, both siRNAs in a single cell would block infection by a heterodiploid virus as well as v120-A/v126-D recombinant because these two types of viruses would be sensitive to one or both siRNAs. Because siRNA120a targets the 5'end of v120-A *env *gene and siRNA126a targets the 3'end of v126-D gene, a virus resistant to both siRNA would harbor chimeric *env *genome with 5' end/upstream region from v126-D and a 3' end/downstream region from v120-A. In addition the breakpoint would have to be between the two siRNA target sequences in the *env *gene.

This study examined the possible influence of siRNA inhibition on HIV-1 replication in a dual infection and how siRNA may be used as a tool to enrich for HIV-1 recombination in specific regions (e.g. *env*) of the HIV-1 genome. Several specific siRNAs were designed and tested. They include a siRNA120a specifically targeting upstream (C1) of *env *in virus v120-A, a primary CXCR4-tropic HIV-1 isolate from a subtype A infected Ugandan, and a siRNA126a specifically targeting downstream (gp41) of virus v126-D, a primary CXCR4-tropic HIV-1 isolate from a subtype D infected Ugandan [23]. Theoretically, any RNA containing either target region will be degraded by siRNA120a or siRNA126a; and only recombinants not containing *env *region upstream of v120-A and *env *region downstream of v126-D can survive, be propagated, and be enriched (Figure 1).

## Results

### Efficiency and specificity of siRNA inhibition on HIV-1 replication

**Figure 2:**
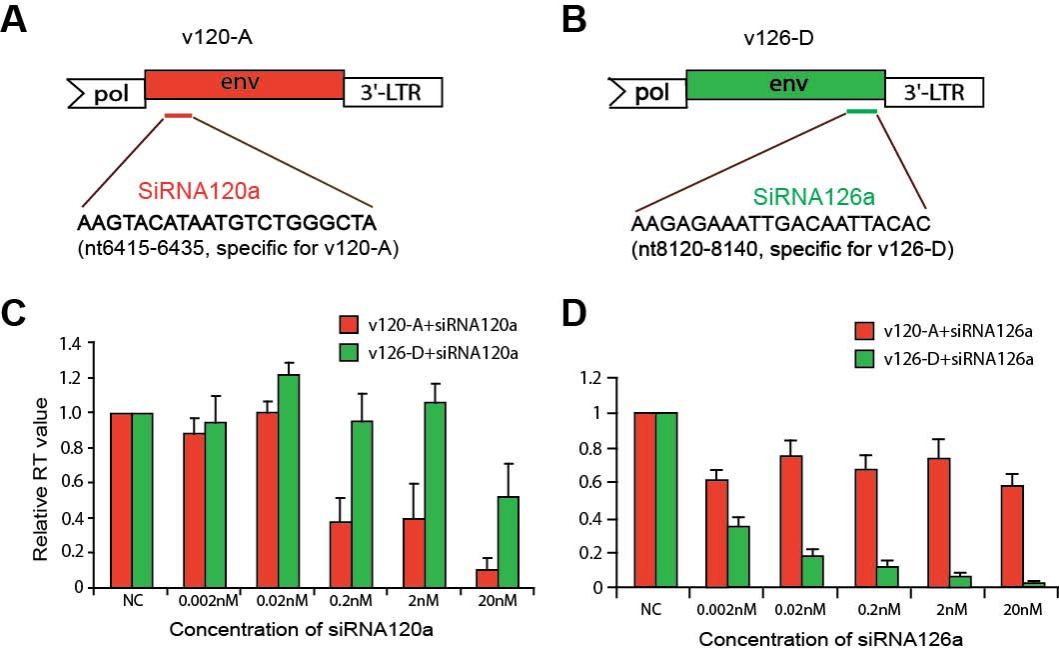
**Efficiency and specificity of siRNA-mediated inhibition of v120-A and v126-D replication**. Panels **A **and **B **illustrate the specificity of siRNA120a for the 5' end of v120-A and of siRNA126a for the 3' end of v126-D. As described in Gao et al. 2008 [23], these siRNAs show specificity based on complete complementarity with the HIV-1 target sequence (nt 6415-6435 for siRNA120a and nt 8120-8140 for siRNA126a) of the specific HIV-1 isolate. The ability of the siRNAs to inhibit v120-A and v126-D is shown in panels **C **and **D**. Panel **C **is a reproduction of a previous experiment presented in Gao et al. [23] and shows the inhibition of HIV-1 v120-A and v126-D by siRNA120a. Panel **D **is showing the specificity of siRNA126a for inhibition of HIV-1 v126-D as opposed to v120-A. Virus production was monitored by RT activity in supernatant at day 5 post-infection and presented relative to the no drug control (NC) (RT values are 1937 and 1852 cpm/ml for v120-A and v126-D, respectively). The IC_50 _value of siRNA120a for inhibition of v120-A was approximately 0.16 nM and 0.021 nM for the IC_50 _of siRNA126a for inhibition of v126-D.

To test the efficiency and specificity of siRNA inhibition on HIV-1 replication, we designed four siRNAs specifically targeting the C1 region of HIV-1 v120-A (subtype A) and two siRNAs specifically targeting the gp41 region of v126-D (subtype D). Both HIV-1 v120-A and v126-D strains were derived from treatment-naive HIV-1-infected pediatric patients in Kampala, Uganda in 1996. The inhibitory activity of all siRNAs was previously tested in U87.CD4.CXCR4 cells with replication competent, primary isolate virus v120-A or v126-D by detecting HIV-1 reverse transcriptase activity at different time points post-infection. As previously reported, we found that siRNA120a inhibited v120-A replication with the greatest efficiency; siRNA120b and siRNA120c were moderately efficient; and siRNA120d lacked significant inhibitory activity. Similarly, siRNA126a showed greater potency against v126-D than did siRNA126b [23]. siRNA inhibition is sequence-specific (Figure 2C and 2D) with modest inhibition of HIV-1 v126-D replication even with siRNA120a at high concentrations (20 nM) (Figure 2C). The lack of substantial inhibition was also observed despite only a 4 nucleotide mismatch between the siRNA126a and the envelope gene of the v120-A target sequence (Figure 2D).

When selecting for siRNA-resistance, often a single nucleotide substitution is sufficient to elicit resistance [27]. When designing different siRNAs to inhibit one versus another HIV-1 strain, a single nucleotide difference may also be sufficient, but this approach requires considerable screening effort. Instead, we analyzed the v120-A and v126-D sequences for sites of high genetic diversity between the two strains and within the *env *region of interest. It was best to maximize mismatches between the strains in order to minimize cross-inhibition and possible miRNA-like repression [28].

**Figure 3:**
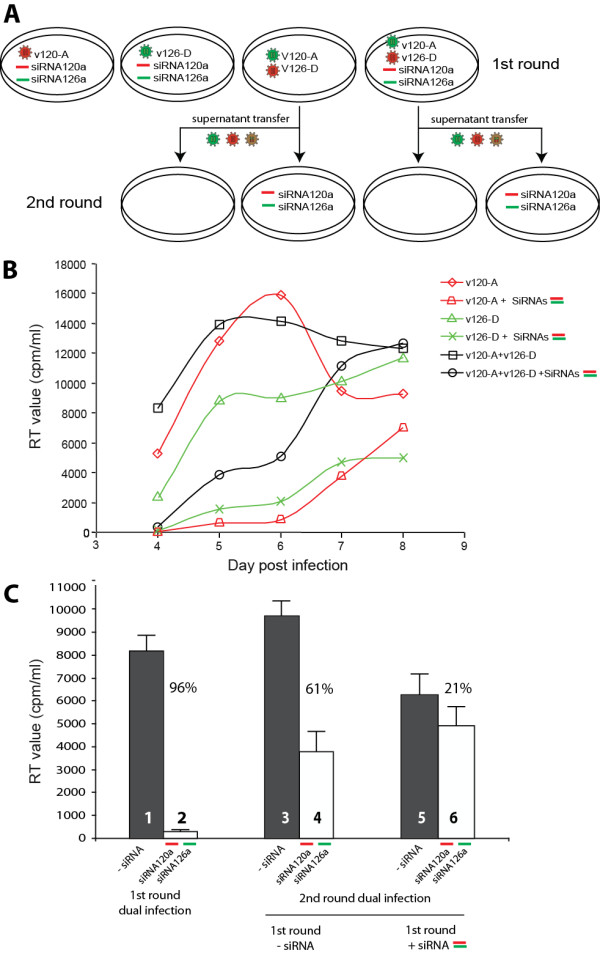
**siRNA inhibition of mono- and dual-infections with HIV-1 v120-A and v126-D**. (**A**) Schematic illustration of the monoinfections or dual infections with or without siRNA treatment for a first round on U87.CD4.CXCR4 cells. The supernatant of this round was monitored at days 4, 5, 6, 7, and 8 for virus production using a radiolabelled RT assay. The RT activity (cpm/ml) over this time course with or without siRNA treatment was plotted in panel **B **(error bars were removed for better viewing). For the second round infection of panel A, virus-containing supernatants from the day 5 dual infections with or without dual siRNA treatment were equalized for RT activity and then added to fresh U87.CD4.CXCR4 cells. RT activity from the supernatant at day 4 from this second round infection and the first round dual infection was plotted in panel **C**. In the first round, a 96% inhibition was observed between dual virus production without (bar 1) versus with dual siRNA treatment (bar 2). When virus from the first round in the absence of siRNA treatment was added to the second round infection, dual siRNA treatment mediated a 61% inhibition (bar 4 versus 3). Finally, infection with siRNA-treated virus from the first round resulted in only a 21% inhibition by siRNAs in the second round (bar 6 versus 5).

In our previous study [23], we characterized the mechanism and kinetics of siRNA inhibition of HIV-1. HIV-1 inhibition by siRNA was greatest at days 4 to 5 with breakthrough starting at day 6. By day 8, virus rebound was apparent, as is the case with monoinfections with v120-A or v126-D treated with siRNAs (Figure 3B). However, this virus rebound was due to a single bolus of siRNA delivered via lipofectamine followed by a slow decay. The virus that rebounded in monoinfections with siRNA treatment is wild type with a very low frequency of mutant virus (mutations in the siRNA target sequence). Although siRNAs select for HIV-1 with mutations in the siRNA target sequence, the selected mutant virus must compete with wild type virus that rebounds as a result of insufficient inhibition after prolonged siRNA treatment.

### siRNA inhibition was overcome by dual infection

We next examined the prolonged inhibitory effect of siRNAs on both mono- and dual-infections (Figure 3A). Monoinfections with v120-A and v126-D in the absence of siRNA treatment obtained the highest levels of RT activity within five to six days; but when treated with specific siRNAs, the inhibition was nearly complete during this same time period (Figure 3B) [23]. The addition of both siRNAs to the monoinfections also resulted in nearly complete inhibition of HIV-1 v120-A or v126-D replication (Figure 3B). Furthermore, complete inhibition of a dual v120+v126 infection (96%) was observed at day 4 with the dual siRNA treatment (Figure 3B and first two bars, Figure 3C). However, dual infection in the presence of both siRNAs resulted in a more rapid rebound in virus replication than observed in monoinfections with the specific inhibitory siRNA (Figure 3B). By day 8, dual siRNA treatment showed minimal inhibition of dual virus production (compare v120+v126 infection +/- siRNAs; Figure 3B). In contrast, there was significantly less rebound at day 8 in virus production with the monoinfections in presence of a single specific siRNA or both siRNAs (Figure 3B). This data suggest that rebound or "escape" from siRNA inhibition was more evident in dual infections than in monoinfections.

Based on the experiments in Figure 3B, one hypothesis suggests that the breakthrough observed in a dual infection and in the presence of both siRNAs may be related to generation of heterodiploid virus (v126-D + v120-A RNA genomes) followed by recombination (Figure 1). A recombination event (v126-D/v120-A) with a breakpoint within *env *and between the siRNA target sequences could render the virus resistant to both siRNAs (Figure 1). To explore this possibility further, viruses produced from dual infections in the presence or absence of siRNA were equalized for RT activity and added to fresh U87.CD4.CXCR4 cells, again in the presence or absence of both siRNAs (Figure 3A). As described earlier, after day 4 post dual infection in the first round, dual siRNA treatment resulted in 96% inhibition, despite a rebound at day 8. In presence of siRNA in the second round at day 4, there was only a 61% inhibition as compared to 96% siRNA inhibition observed in the first round dual infection at day 4 (compare bars 4 vs 3 and 2 vs 1, Figure 3C). In the second round, this reduced virus inhibition (with virus innoculum from first round dual infection) is likely related to the production of heterodiploid and recombinant virus in the first round (see below). Again, the heterodiploid virus has the potential of recombining in *env *such that the resulting virus is resistant to both siRNAs targeting different ends of the *env *gene. When siRNA-treated virus from the first round was used to infect fresh cells in the second round, there was only a 21% inhibition by the dual siRNA treatment as compared to 96% observed in the first round (compare bars 6 vs. 5 and 2 vs. 1, Figure 3C). Since siRNA treatment from the first round had the potential to already select for recombinants resistant to siRNAs, it is not surprising that this population could be further enriched in the second round.

### HIV-1 recombinants were greatly enriched by siRNAs treatment

In relation to the observations in Figure 3, the "breakthrough" of HIV-1 replication in the presence of potent dual siRNA treatment is likely due to generation of HIV-1 intersubtype recombinants between the subtype A v120-A and subtype D v126-D. Alternatively, siRNA may have selected for v120-A and/or v126-D with nucleotide substitutions in the siRNA120a and siRNA126a target sequences, respectively. If the first hypothesis is correct, siRNA120a and siRNA126a treatment will only enrich those recombinants containing upstream *env *(C1) of v126-D and downstream *env *(gp41) of v120-A, which can escape siRNA targeting and degradation. The infected U87.CD4.CXCR4 cells were harvested at day 5 post-infection, and HIV-1 DNA were extracted for PCR amplification employing subtype-specific oligonucleotide primers to detect, amplify, and quantify *env *recombinants in HIV-1 dual infections [12,13,17]. Here, we used subtype-specific primers (ESD1 and ESA2) to amplify envelope fragment from 5' v126-D/3' v120-A *env *recombinants and conserved primers (EAD1 and EAD2) to amplify both v126-D and v120-A *env *genes. The specificity of the primer sets to detect and amplify *env *genes of the parental and recombinant viruses was fully tested (data not shown).

**Figure 4:**
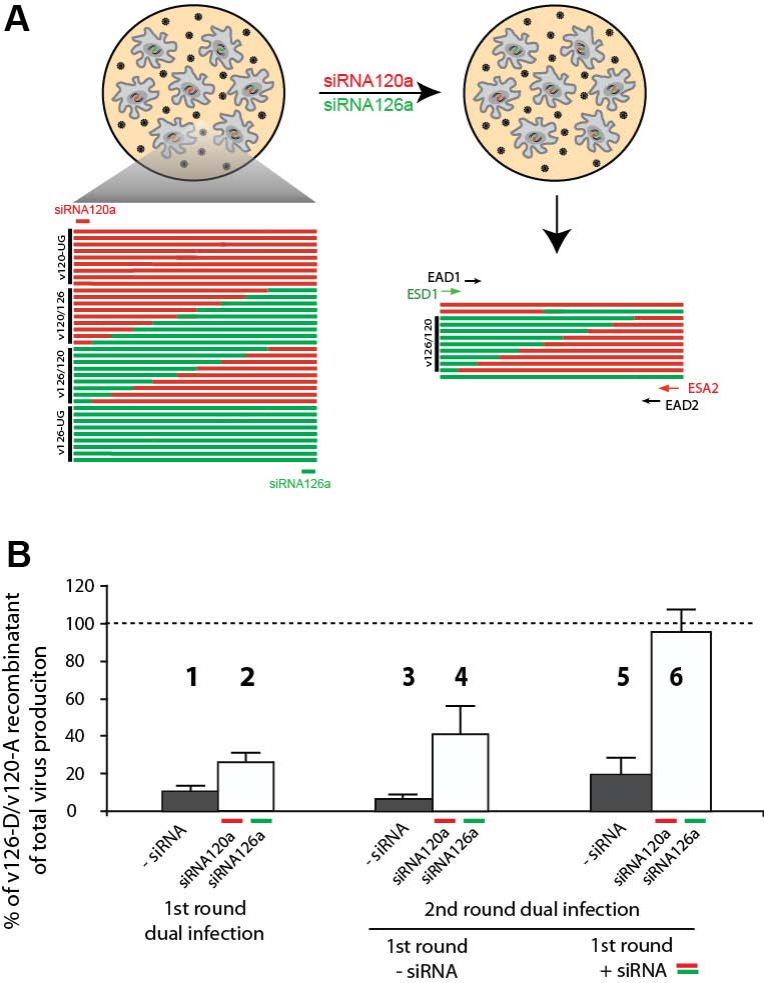
**Estimating the frequency of v126-D/v120-A recombination with or without siRNA enrichment using semi-quantitative PCR**. Schematic describing the siRNAs enrichment of v126-D/v120-A recombinants and the PCR strategy designed for their detection and quantification is shown in panel **A**. Virus 126-D and virus 120-A *env *specific primers were used to PCR amplify the *env *recombinant genes alongside conserved *env *primers amplifying all *env *genes (see Materials and Methods). The viruses produced in the first and second round infections (bars 1 through 6 in Figure 3C) were used as templates for this PCR of the v126-D/v120-A *env *or all of the *env *genes in the virus (i.e. v120-A + v-126-A + v126-D/v120-A + v120-A/v126-D). A PCR control involved PCR amplification of 10-fold dilutions of v120-A and v126-D *env *DNA in a DNA vector construct. Panel **B **shows the percentage of v126-D/v120-A recombinants in the total virus measured by semi-quantitative PCR.

The percentage of siRNA-resistant recombinants (between the siRNA target sequences in *env*) was then determined by the fraction of recombination-specific ESD1-ESA2 PCR product divided by the parental/recombinant (total) EAD1-EAD2 PCR products. In the absence of siRNA treatment, the percentage of 126-D/120-A *env *recombinants (11%; bar 1 in Figure 4B) generated from a dual infection was low as expected [13]. It is important to note that the frequency of recombination was monitored a day subsequent to the measurement of maximal virus inhibition. Breakthrough replication was already evident at day 5 in the first round of dual infection in the presence of siRNAs (only 72% inhibition at day 5 versus 96% at day 4). As a consequence, it is not surprising to observe a possible selection of 126-D/v120-A *env *recombinants in the siRNA-treated first round dual infection (26%; bar 2, Figure 4B). When untreated virus from the first round is used to infect fresh cells, the level of recombinants is only 6.7% in absence of siRNA. Interestingly, there was an increase in 126-D/v120-A *env *recombinants in the siRNA-treated second round infection (with virus from untreated first round) as compared to the recombinants detected in the siRNA-treated first round (compare 41% of bar 4 to 26% of bar 2, Figure 4B). Finally, over 96% of the virus harbored a 126-D/120-A *env *, if the dual virus infections were treated with the siRNA120a and siRNA126a for two rounds (bar 6, Figure 4B). It is important to note that HIV-specific siRNAs do not target the reverse transcription process, but only inhibit subsequent or during mRNA synthesis [23]. Thus, preferential replication of virus harboring the 126-D/120-A *env *represents an enrichment of recombinants resistant to siRNAs rather than "escape" from siRNA inhibition during transcription. This siRNA-mediated enrichment to 96% recombinants could have originated from the 11% 126-D/120-A *env *recombinants of the replicating virus population generated in the absence of siRNAs.

### Identifying of 126-D/120-A *env *recombinants and mapping recombination breakpoints

**Figure 5:**
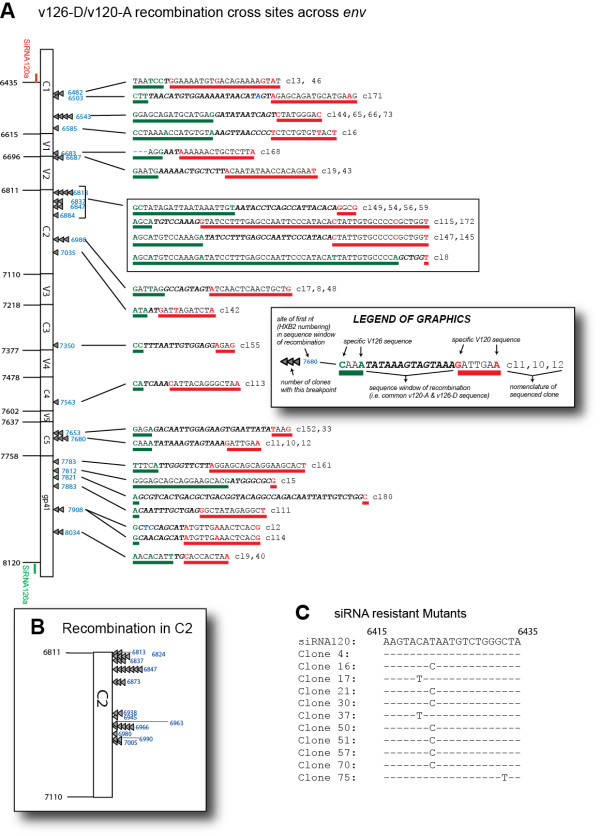
**Mapping the v126-D/v120-A recombination sites in *env *that led to siRNA resistance**. **(A) **In the sample with two rounds of dual siRNA120a and siRNA126a treatment, 39 of 50 sequenced clones were v126-D/v120-A recombinants with recombination breakpoints mapping to region between the two siRNA target sequences (nt6435 to 8120). The "Legend of Graphics" provides a description of (1) site of the first nt in a sequence window for a recombination breakpoint, (2) the nt sequence with v126-D specific sequences immediately preceding the window of recombination, (3) the actual window of recombination with identical v126-D and v120-A sequence, and (4) the nt sequence with v120-A specific sequences immediately following the window of recombination. **(B) **The fine mapping of the recombination breakpoints in the *env *C2 region was determined by first PCR amplifying the *env *PCR product with nested primers (specific for v126-D and v120-A in the C2 region), cloning these products into pCR XL TOPO vector (Invitrogen), and then sequencing 33 clones. **(C) **Eleven of 50 clones did not contain a v126-D/v120-A but nine of these were v120-A with a specific mutation in siRNA120a target sequence (nt6415 to 5435, except clone #4). Two of the 11 were v120-A/v126-D recombinants which also harbored a mutation in the siRNA120a target sequence.

To map the site of intersubtype recombination, 30 *env *clones from the untreated infections and 50 from the two rounds siRNA-treatment were sequenced and aligned. These analyzes revealed that only 1 out of 30 clones was a v126-D/v120-A recombinant in the untreated infections. In this untreated sample, the identification of twenty-two v120-A clones and only 7 v126-D clones is consistent with the increased fitness of v120-A (or A15-UG) virus over v126-D (or D14-UG) virus in dual virus competition studies [29]. In the siRNA-treated samples, the majority or 39 *env *clones were v126-D/v120-A recombinants; 2 were unexpectedly v120-A/v126-D recombinants; and 9 were v120-A (Figure 5).

Sequence analyses revealed that recombination breakpoints were scattered throughout the *env *gene, but with more recombination sites appearing in conserved regions and with a "hotspot" in C2 (Figure 5A). Only 3 of the 39 126-D/120-A clones (or 8%) had a recombination breakpoint in the hypervariable regions (V1, V2, V3, V4, and V5), even though these 440 nucleotides account for 26% of the sequence in this *env *segment (targeted by siRNAs). The discussion will highlight how increased sequence conservation enhances but is not a requirement for intersubtype HIV-1 recombination.

The hotspot in C2 has been previously described for recombination between HIV-1 v120-A and v126-D [12,13,17]. However, in those studies, this C2 hotspot was observed in dual infections lacking siRNA enrichment and also in a single-cycle recombination system without selection for replication-competent intersubtype recombinant virus [12,13,17]. As described below, maintenance of a C2 hotspot for v126-D/v120-A recombination suggests that siRNA treatment does not alter the distribution of recombination breakpoints. In previous studies as in these analyzes, the frequency of intersubtype v126-D/v120-A recombination after multiple rounds of replication was less than 5% in the absence of siRNA selection/enrichment [13]. As a consequence, fine mapping of recombination in the C2 hotspot was not feasible. However, with siRNA selection, about 80% of the replicating virus population harbors a breakpoint within the *env *gene, and over 33% of these have a breakpoint in C2. To further map the C2 breakpoints, the C2 region was PCR amplified with an upstream v126-specific primer paired with downstream v120-specific primer. The PCR products were cloned and sequenced from thirty-three v126-D/v120-A C2 *env *clones. These analyses revealed 12 unique breakpoints in a 300 nt sequence in C2 (Figure 5B). Based on the sequence identity between the C2 regions of v126-D and v120-A, we could map breakpoints to 22 windows varying from 1 to 25 nt in length. A window for possible recombination is defined by identical v120-A and v126-D sequence flanked by nucleotide substitutions, between the two strains, (e.g. 5' and 3' of the window of sequence identity) (see legend of Figure 5A). A specific, more defined recombination hotspot in C2 (nt 6811-6873) has been characterized, but this involved mapping breakpoint from a single cycle recombination system using defective virus particles. It is important to note that the use of siRNAs in a dual infection/recombination system would only enrich for breakpoints that generated functional *env *glycoproteins and replicating virus.

As described earlier, another form of escape from dual siRNA inhibition in this system may involve mutations at the siRNA target sequence rather than escape through recombination. As described earlier, after 2 rounds of dual siRNA selection, 39 of 50 sequence clones harbored breakpoints between the siRNA120a and siRNA126a that target 5' end of v120-A and 3' end of v126-D, respectively. The remaining 11 clones had a complete v120-A *env *gene (9) or v120-A/v126-D recombinant *env *gene. However, 10 of these clones contained single nucleotide substitutions in the siRNA120a target sequence in v120-A *env*. These single nt substitutions, the most predominant being T to C at position 6422 (HXB2 numbering), were likely associated with siRNA120a resistance (Figure 5C). As described below the predominance of siRNA120a-resistant v120-A as opposed to siRNA126a-resistant v126-D is likely due to the increased replication of v120-A over v126-D in the dual infections (observed in the absence of siRNA) [29].

### Continual passaging of virus progeny in the presence of siRNAs

Dual infection and A/D recombination occur at much higher frequency (>5%/1000 nt or up to 15-20% between the siRNA target sequences) [13] than the highest point mutation frequency (<0.1% within the siRNA target sequences based on 3.4 × 10^-5 ^mutations per nt per cycle)[30] in the absence of selection. These findings would suggest a greater abundance of replicating A/D recombinants with siRNA-resistance than HIV-1 harboring siRNA point mutations immediately after dual infection. However, we have shown in our previous studies that v120-A (or A15-UG) was significantly more fit than v126-D (or D14-UG) which might imply that even the v120-A/v126-D HIV-1 recombinants may be less fit than parental v120-A. To explore this possibility, we serially passaged the original dual infections (performed in triplicate) in the presence of both siRNAs. By passage 8, we could not detect A/D recombinants by PCR, and all 20 sequenced clones were indeed v120A with a single T to C mutation at position 8 in the siRNA target sequence (the same as the mutant clone dominant in Figure 5C, i.e. clone16, 21, 30, 50, 51, 57, and 70). Based on these findings it appears that v120-A, even with these siRNA-resistant mutations, was more fit that siRNA-resistant A/D recombinants and obviously more fit than v126-D with any siRNA target site mutations (which never appeared in the virus population).

## Discussion

Even though intersubtype recombinants are evident in humans co- or super-infected with two or more different HIV-1 isolates [31-35], the frequency and survival of intersubtype HIV-1 recombinants are highly variable during disease. The few studies on de novo emergence of intersubtype recombination in vivo reflect the difficulties of identifying dual or super-infections at time of actual occurrence as well as the careful follow-up required to identify possible recombinants. As a consequence, analyses of HIV-1 recombination are quite common in vitro but are limited as a model for various reasons. Based on in vitro studies, the frequency of retroviral recombination within the 9.7 kilonucleotides of HIV-1 genome fluctuates between three and thirty recombination events per round of replication and is highly dependent on (i) cell type [36], (ii) the use of primary HIV-1 isolates [12,13,17] versus defective strains [14-16], (iii) sequence identity between different HIV-1 strains/genomes [12,13], and (iv) selection of all recombinants [14-16] or only those that are replication competent [12,13,17,37]. Many studies including those of our group have helped to elucidate various factors driving intersubtype recombination following infections with a defective retrovirus harboring a heterodiploid genome [12-16,36,37]. The most striking observations may relate to an obvious increase in intersubtype recombination in genomic regions with the highest sequence identity [12]. Although this might be expected, we also observed "hotspots" for intersubtype recombination breakpoints that are less dependent on sequence identity and may be more related to the mechanism(s) of strand transfer during reverse transcription [13,38].

In vitro models likely identify the correct progenitors of intersubtype recombinants, but the final composition of intersubtype recombinants in a dually infected patient may reflect other factors, which include the obvious requirement of virus replication in the face of different host and immune selective pressures. To understand which intersubtype recombination breakpoints can lead to replication competent virus, we had to develop a system to enrich for intersubtype HIV-1 recombinants in tissue culture. In past studies, we have examined intersubtype recombination through a dual infection of cell lines or primary human cells with two or more primary HIV-1 isolates [12,13,17]. In the first round of dual infection, the frequency of co-infected cells (with two different viruses) reflects the initial multiplicities of infection (MOI). For example, in a flask with 100,000 cells, an MOI of 0.01 virus A and 0.01 virus B (i.e. 1 virus per 100 cells) would result in approximately 10 cells being co-infected with both virus A and B. However, previous reports suggest that with two identical virus strains (aside from a marker) the frequency of dual infection is often higher than expected from random virus-cell interactions [39]. In these co-infect cells, Hardy-Weinberg equilibrium and an assumption of equal packaging of both RNA genomes would predict that half the virus would be heterodiploid. As virus titer of both A and B increased during multiple rounds of replication, so would heterodiploid virus production, but eventually all susceptible cells are exhausted for infection in the culture. Thus, parental viruses (e.g. A and B) always dominate a dual infection and basically obscuring the characterization of replication-competent intersubtype HIV-1 recombinants, i.e. present at very low levels. Adding the complexity to this system, recombinants are only generated following a de novo infection with a heterodiploid A+B virus. Even though the frequency A/B cross-over event during reverse transcription can be as high as 50% over a 9.7 nt genome, less than 10% of these resulting intersubtype HIV-1 recombinants survive due to generation of defective virus or simply being unable to compete with the parental strains [13].

To focus our analyses on intersubtype recombination, we developed a system to selectively inhibit parental virus replication in a dual infection and as consequence, to enrich for only intersubtype HIV-1 recombinants. This system first involved the design and testing of virus-specific siRNA that would not only inhibit replication of a single parental virus, but would also enrich for intersubtype recombination in a specific HIV-1 genomic region. For the purposes of this study, siRNA120a selectively inhibited a subtype A primary HIV-1 isolate (v120-A) by targeting a 5' sequence in the HIV-1 *env *genes whereas a 3' *env *sequence was targeted by siRNA126a to specifically inhibit a subtype D primary HIV-1 isolate (v126-D). This inhibition of HIV-1 replication by siRNAs is due to the targeting and degradation of newly transcribed HIV-1 mRNA. We and others have recently shown that incoming HIV-1 genome RNA is protected by the HIV-1 core proteins and cannot be degraded by the RISC-siRNA complex [23,40]. Destablization of the core with the exogenous addition of TRIM5α will increase core dissociation, increase RISC-siRNA access, and result in HIV-1 RNA degradation. However, this process is not activated in normal conditions of human cell lines [23]. Based on siRNA degradation of only HIV-1 mRNA and following reverse transcription, our dual siRNA treatment would not influence the retroviral recombination mechanism but only select for those intersubtype D/A recombinants with breakpoints that occurred at a frequency of >5%/1000 nt in the *env *gene [13] and up to 15-20% between the siRNA target sequences. These D/A *env *recombinants could be propagated and enriched in culture considering they are resistant to the siRNA inhibition of HIV-1 mRNA transcription, again at a step subsequent to reverse transcription and integration. As illustrated in Figure 4B, D/A *env *recombinant virus became the majority of replicating virus when treated with the two siRNAs during two rounds of propagation. In absence of this siRNA enrichment/selection, D/A *env *recombinant virus represented only 6.7% of the replicating virus population, which was dominated by the parental v120-A and/or v126-D virus.

The frequencies of recombination in these propagated dual infections were initially estimated by PCR amplification using isolate-specific oligonucleotide primers. However, due to non-specific amplification, these recombination frequencies were likely over-estimates. To determine a more accurate level of recombination and more importantly, to map the site of recombination, the dual infections propagated in the presence or absence of siRNAs were PCR amplified with conserved *env *primers. Cloning and sequencing of these *env *products revealed that D/A recombinants could hardly be detected in the absence of siRNAs, but that dual siRNA treatment resulted in 39 v126-D/v120-A recombinants in 50 *env *clones. Interestingly, the remaining clones were v120-A or v120-A/v126-D *env *genes, and 10/11 harbored mutations in the siRNA120a target sequence. We suspect that the mutations in this target sequence conferred resistance to the siRNA120a and as consequence would be resistant to both siRNAs. Predominance of v120-A with siRNA120a resistant mutations as opposed to v126-D with siRNA126a mutations likely relates to the increased fitness of v120-A over v126-D in these dual infections [29]. In fact, we did not observe v126-D/v120-A recombinants after eight rounds of dual siRNA selection, but instead v120-A dominated with a single mutation in the siRNA120a target sequence. We are now determining how dual infections with viruses of equal or different fitness might influence (1) the frequency of recombination, (2) the rate of intersubtype recombinant viruses in the presence of dual siRNA treatment, and possibly, (3) the sites of recombination breakpoints.

Our previous studies indicated that intersubtype recombination breakpoints were scattered across conserved regions, i.e. C1, C2, and C3, using a single cycle assay [12,13]. As mentioned earlier, the multiple cycle assay in the absence of siRNA enrichment results in a very low level of recombinants co-circulating with the parental strains in culture. As a result, we suspect that the pattern of v126-D/v120-A recombination in our previous multiple-cycle/dual infection assays may be the result of some re-sampling of recombinant clones [12]. In addition, obtaining replication-competent recombinants from the dual infection was less likely in the absence of siRNA enrichment due to the continuous generation of both defective and replication-competent recombinants and the ongoing parental strain replication. In the study presented herein, use of dual siRNA treatment inhibited the replication of parental strains, and only 126D/120A recombinants were likely to survive two rounds of propagation. In addition, we examined recombination sites across most of the *env *gene (~1700 nt) (this study) as opposed to just the C1-C4 regions (~1100 nt) [12]. Nearly identical numbers of recombination breakpoints were identified in the C2 region as compared to other C1-C4 regions regardless of system, i.e. 45% 126D/120A recombination breakpoints in C2 with single cycle system versus 46% with multiple cycle [12] and 48% with multiple cycle in the presence of siRNA enrichment (this study). The differences relates to the positioning of the breakpoints in C2. Replication-competent 126D/120A recombinant viruses appeared to harbor more breakpoints near the V2/C2 junction than those from single cycle assays. Due to the siRNA enrichment, we could now perform more careful mapping of the 126D/120A breakpoints in the C2 region of *env*. It is now quite clear that recombination in C2 maps to two specific regions, nt positions 6813-6873 and nt positions 6938-7005. The C2 region is 299 nt in length and yet, aside from these 60 and 67 nt segments, the remaining 172 nt have nearly no recombination breakpoints. These findings clearly indicate that pattern of breakpoints in intersubtype HIV-1 recombinants is shaped by both the reverse transcription process and during subsequent selection for replication competent virus.

Although numerous selective forces could influence selection of these intersubtype HIV-1 recombinants within a host, several studies have now shown a clear overlap in the recombination breakpoints derived from intersubtype HIV-1 recombinants generated in tissue culture with those identified in unique and circulating intersubtype recombinant forms (URFs and CRFs) found in the HIV-1 epidemic [12,17,38]. Considering that HIV-1 intersubtype recombinants represent approximately 20% of all infections, vaccines and new drug therapies could be designed based on a better understanding of strand transfer mechanisms during reverse transcription and the actual breakpoints that give rise to functional, replication competent virus. It is also apparent that intersubtype HIV-1 recombination, following a dual infection or superinfection of already infected individual, could lead to rapid immune escape. A simple comparison of our A/D env sequences with HIV-1 epitope maps, restricted by common HLA alleles (http://www.hiv.lanl.gov/content/immunology/maps/ctl/gp160.html), revealed that 30/39 of the clones had recombination breakpoints within immunodominant epitopes.

## Conclusions

In summary, we have developed a method to rapidly enrich for HIV-1 recombinants by blocking each of two parental HIV-1 isolates in a dual infection with strain-specific siRNAs. Using this approach, we could easily detect, map, and characterize intersubtype breakpoints in the HIV-1 *env *gene. Nearly 33% of all v126-D/v120-A recombination sites in *env *mapped to two ~60 nt sequence in the C2 regions (0.27 recombination sites/nt) whereas the remaining 67% were scattered 1700 nt C1-to-gp41 region of *env *(0.05 recombination sites/nt). It was paramount to prove that our methods used to enrich recombinants generated a similar pattern of intersubtype A/D breakpoints as those previous observed in our A+D dual infections, but in absence of siRNA selection. If siRNA enrichment skewed the distribution of recombination breakpoints, this methodology would not have been useful as a model of in vivo intersubtype recombination. This system now provides a high population of replication-competent intersubtype recombinants (~80%) whereas dual infection without siRNA selection generates less than 2% recombinants[13], the majority of which are not replication competent [37]. Dual infection coupled with a siRNA enrichment/selection is now being used to rapidly diversify HIV-1 gene segments and even whole genomes. The replication competent, recombinant viruses can be used for heterogeneous vaccine constructs or a swarm of divergent viruses for drug inhibition/resistance studies.

## Methods

### Cell culture

PBMCs from HIV-1 seronegative donors were separated from heparinized blood by Ficoll-Paque density centrifugation and cultured in RPMI-1640 medium (Mediatech, Inc.) supplemented with L-glutamine, 10% fetal bovine serum (FBS, Mediatech, Inc.), 10 mM HEPES buffer, penicillin (100 U/ml), streptomycin (100 μg/ml), 1 U of phytohemagglutinin/ml, and 1 ng of interleukin-2 (Gibco)/ml. The cells were suspended (2 × 10^6 ^cells/ml) and grown for 3 days in culture before use in virus propagations. U87.CD4.CXCR4 and U87.CD4.CCR5 cell lines were obtained from the AIDS Research and Reference Reagent Program and grown in Dulbecco's modified Eagle's medium (DMEM, Cellgro) supplemented with 15% FBS, penicillin and streptomycin, puromycin (1 μg/ml) and G418 sulfate (1 mg/ml) at 37°C and 5% CO_2_.

### Viruses

v120-A (subtype A, CXCR4 tropic) and v126-D (subtype D, CXCR4 tropic) were obtained from two treatment-naive HIV-1-infected pediatric patients in Kampala, Uganda in 1996. The viruses were isolated and propagated by co-culturing PBMCs from the patients and from healthy donors. Prior to co-culture, PBMC were pre-stimulated with PHA and cultured with IL-2 as described above. TCID_50 _assays (tissue culture dose for 50% infectivity) were performed to determine virus titer[41]. Titers were expressed as infectious units per milliliter.

### SiRNAs preparation

Twenty-one-nucleotide dsRNAs were chemically synthesized as 2' bis(acetoxyethoxy)-methyl ether-protected, desalted and duplexed oligonucleotides by Dharmacon (Lafayette, Colo.). Six siRNAs (siRNA120a, siRNA120b, siRNA120c, siRNA120d, siRNA126a, and siRNA126b) were designed according to both the manufacturer's recommendations and to the *env *sequences of v120-A and v126-D. These siRNAs were previously utilized in another study to characterize the mechanism(s) of siRNA inhibition of HIV-1[23]. According to HXB2 numbering, siRNA120 is located at nt 6415-6435 or nt 6400-6420 in the C1 region of the *env *gene in v120-A, and siRNA126 is located at nt 8120-8140 or nt 8604-8624 in gp41 coding region of the *env *gene in v126-D. Control siRNAs vary from 1 to 5 nucleotide differences. The efficiency of siRNAs were tested as described previously [23].

### SiRNA inhibition efficiency on HIV-1 dual infection

One × 10^5 ^U87.CD4.CXCR4 cells were plated in 24-well plates, and 48 hours later, infected with 0.1 multiplicity of infection (MOI) of HIV-1 v120-A or v126-D, or with both viruses. The supernatants were harvested from different time points postinfection for monitoring the virus production. After the initial dual infection, the virus-containing supernatant from day 5 of the dual infection was used to infect fresh, untreated U87.CD4.CXCR4 cells or cells transfected with 20 nM of siRNA120a and/or siRNA126a by Lipofectamine 2000 (Invitrogen) as previously described [23]. Supernatant from this second round infection was replaced with fresh media after 6 hours incubation. Supernatant was then collected at day 4 to 10 post infection. The supernatants from the first round infection (in the presence or absence of siRNAs) were then used for a second round infection with the same protocol described for the first round. Virus levels in the supernatants for all these experiments were monitored by RT assay as previously described[41].

### Semi-quantitative PCR detecting of the frequency of HIV-1 recombinants

Isolate-specific primers were designed for amplification of 126-D/120-A *env *recombinants: ESD1 (sense, nt6407-6426 in HXB2) and ESA2 (antisense, nt8133-8114). Two conserved primers, i.e. EAD1 (sense, nt6427-6444) and EAD2 (antisense, nt8082-8064), were used to amplify both HIV-1 recombinants and their parental viruses. Proviral DNA was extracted from the infected U87.CD4.CXCR4 cells using the QIAamp DNA blood kit (Qiagen) and serially diluted (1:10, 1:100, 1:1000, 1:10000) for semi-quantitative PCR as previously described[23]. PCR products of HIV-1 recombinant and overall HIV-1 DNA were analyzed on agarose gel for measuring the frequency of HIV-1 recombinants in the virus population.

### Sequencing and phylogenetic analyses

The above PCR-amplified *env *products were separated on agarose gels and then purified using the Gel Purification Kit (Qiagen), followed by ligation into pCR XL TOPO vector. *Env*-containing pCR XL TOPO clones were sequenced with EAD1, E80, and EAD2 (Davis sequencing). The *env *recombinant sequences and parental virus sequences were aligned using the CLUSTAL X v.1.63b program[42]. After sequencing of the recombinant clones, breakpoints were identified by visual inspection. Accuracy of breakpoint location is related to the length of the region between mismatches.

## Abbreviations

siRNA: small interfering RNA.

## Competing interests

The authors declare that they have no competing interests.

## Authors' contributions

YG designed the study, performed the experiments and drafted the manuscript. MA and KNN performed generation of v126-D/v120-A *env *recombinants through siRNA treatment, and subsequent screening and sequencing. HB helped with analysis of recombinants and determination of breakpoints. DM and AA helped with virus propagation assay. EJA provided overall supervision for the project, secured funding, and helped write the manuscript. All authors read and approved the final manuscript.
